# Estimating the effect of pretreatment loss to follow up on TB associated mortality at public health facilities in Uganda

**DOI:** 10.1371/journal.pone.0241611

**Published:** 2020-11-18

**Authors:** Stella Zawedde-Muyanja, Joseph Musaazi, Yukari C. Manabe, Achilles Katamba, Joaniter I. Nankabirwa, Barbara Castelnuovo, Adithya Cattamanchi

**Affiliations:** 1 The Infectious Diseases Institute, College of Health Sciences, Makerere University, Kampala, Uganda; 2 Division of Infectious Diseases, Department of Medicine, Johns Hopkins University School of Medicine, Baltimore, Maryland, United States of America; 3 Department of Medicine, School of Medicine, Makerere University College of Health Sciences, Kampala, Uganda; 4 Division of Pulmonary and Critical Care Medicine and Center for Tuberculosis, University of California San Francisco, San Francisco, California, United States of America; University of Pretoria, SOUTH AFRICA

## Abstract

**Introduction:**

Tuberculosis (TB) mortality estimates derived only from cohorts of patients initiated on TB treatment do not consider outcomes of patients with pretreatment loss to follow-up (LFU). We aimed to assess the effect of pretreatment LFU on TB-associated mortality in the six months following TB diagnosis at public health facilities in Uganda.

**Methods:**

At ten public health facilities, we retrospectively reviewed treatment data for all patients with a positive Xpert^®^MTB/RIF test result from January to June 2018. Pretreatment LFU was defined as not initiating TB treatment within two weeks of a positive test. We traced patients with pretreatment LFU to ascertain their vital status. We performed Kaplan Meier survival analysis to compare the cumulative incidence of mortality, six months after diagnosis among patients who did and did not experience pretreatment LFU. We also determined the health facility level estimates of TB associated mortality before and after incorporating deaths prior to treatment initiation among patients who experienced pretreatment LFU.

**Results:**

Of 510 patients with positive test, 100 (19.6%) experienced pretreatment LFU. Of these, we ascertained the vital status of 49 patients. In the six months following TB diagnosis, mortality was higher among patients who experienced pretreatment LFU 48.1/1000py vs 22.9/1000py. Hazard ratio [HR] 3.18, 95% confidence interval [CI] (1.61–6.30). After incorporating deaths prior to treatment initation among patients who experienced pretreatment LFU, health facility level estimates of TB associated mortality increased from 8.4% (95% CI 6.1%-11.6%) to 10.2% (95% CI 7.7%-13.4%).

**Conclusion:**

Patients with confirmed TB who experience pretreatment LFU have high mortality within the first six months. Efforts should be made to prioritise linkage to treatment for this group of patients. Deaths that occur prior to treatment initation should be included when reporting TB mortality in order to more accurately reflect the health impact of TB.

## Introduction

Tuberculosis (TB) is currently the world’s leading cause of death from a single infectious cause. The World Health Organization (WHO) End TB Strategy seeks to reduce TB incidence to less than 10 per 100,000 people and to decrease the proportion of patients dying from the disease to less than 5% by 2035 [1]. However, in 2018, TB caused an estimated 1.5 million deaths globally, including 250,000 deaths among persons living with HIV [2]. This represents an estimated case fatality ratio (CFR) of nearly 15% among all patients with TB and 30% among patients co-infected with HIV.

Although the majority of TB-associated mortality occurs in sub-Sharan Africa (SSA) its accurate measurement is difficult due to lack of vital registration systems in most countries. Mortality estimates in SSA are subsequently derived indirectly by multiplying estimated TB incidence by case fatality rates (CFR) recorded within established cohorts of patients enrolled in treatment programs [3, 4]. Such indirect measures of TB mortality do not take into consideration mortality among patients diagnosed with TB who are lost from care before initiating TB treatment (pretreatment loss to follow-up), and in SSA it is estimated that, up to 38% of patients diagnosed with TB do not initiate TB treatment [5]. Mortality estimates that exclude these patients may therefore under-represent actual mortality from the disease.

In 2018, Uganda reported an estimated 4000 TB-associated deaths including 1500 deaths among persons living with HIV, representing a CFR of 8% (12.5% among PLHIV). In the same year, an estimated 20% of patients experienced pretreatment loss to follow-up (LFU) [6, 7]. In other sub-Saharan African settings, high rates of mortality have been reported among these patients, the majority of which occur outside the healthcare system [8, 9]. In this study, we aimed to quantify mortality among patients diagnosed with TB who experienced pretreatment LFU and to characterize the effect of pretreatment LFU on TB mortality estimates at public health facilities in Uganda.

## Methods

### Study setting and population

Ten health facilities each from a different district were included in this study. The health facilities consisted of including three primary care facilities (HC IV), four district hospitals and three tertiary referral hospitals and were selected to represent the different levels of the public health system. All health facilities have onsite GeneXpert testing services and together serve about 3.5 million people. All health facilities use the standardized national paper-based system for recording patients with signs and symptoms of TB (TB presumptive registers), the results of TB laboratory results (TB laboratory registers), and patients started on TB treatment (TB treatment registers). Additionally all patients initiated on TB treatment within a district (which is typically made up of 20–40 health facilities) are recorded in a district TB register.

We included patients aged ≥15 years with drug susceptible TB, defined as an Xpert^®^MTB/RIF test result positive for TB but negative for rifampin resistance, between January 1^st^ to June 30^th^ 2018. We excluded patients who had sputum samples referred from other health centers for Xpert testing. Eligible patients were identified through review of laboratory registers at the 10 study sites.

### Data collection

We extracted demographic (age, gender) and clinical (HIV status) data from TB laboratory and treatment registers. We also used the TB treatment register to identify patients who did and did not initiate treatment within two weeks of diagnosis despite having a positive TB test recorded in the laboratory register. For patients who initiated TB treatment, we assessed six-month mortality by reviewing treatment outcome data reported in TB treatment registers. For patients who did not initiate TB treatment or whose treatment outcome recorded as lost to follow-up or not evaluated, we checked health facility and district TB treatment registers for up to 3 months after diagnosis. We used the district TB number assigned at the start of treatment to track patients who were initiated on TB treatment across registers. For patients who experienced pretreatment LFU, we used their name, age, sex and residence to track them across registers. If a treatment record was found in either of these registers, we used the treatment outcome reported in the register to assess mortality. If no treatment record was found in the health facility or district TB registers, we conducted phone calls and/or home visits to ascertain TB treatment status and vital status. Home visits were carried out by research nurses paired with health facility staff who had familiarity with the local terrain, spoke the local dialect and had prior experience with patient tracing activities. For patients whose phone numbers were available in the TB registers, the health facility staff called to inquire about the location of the home. For patients who had no recorded phone numbers or whose phone numbers were not functional, health facility staff inquired from local residents about the location of the village. Once in the village, health facility staff approached the local authorities to inquire about the person being traced. In order to protect patient privacy, health facility staff did not reveal the reason for their visit until the patient or their family had been identified and confidentiality could be maintained (S1 Fig). A trained research nurse who accompanied health facility staff on all home visits administered a short questionnaire to patients found alive after obtaining informed consent to inquire about TB treatment status, ART use (if HIV-positive) and previous history of TB disease. Patients who had not yet initiated TB treatment were referred to health facilities. If a patient could not be located, health facility staff inquired about the patient’s current vital status from neighbors.

### Outcome definition

For this study, TB-associated mortality was defined as death from any cause during the six months following TB diagnosis.

### Statistical analyses

We used descriptive statistics to summarize patient demographic and clinical characteristics. Among patients who did and those who did not have their vital status ascertained, proportions were compared using chi-square test. The cumulative incidence of mortality at six months after TB diagnosis among patients who did and did not experience pretreatment LFU was estimated using the Kaplan-Meier method. We used log-rank test to compare survival probability between the two groups. We calculated the cumulative incidence of mortality among patients who did and did not experience pretreatment LFU stratified by HIV status, sex, and age-group (15–24, 25–34, 35–44, ≥45). Risk ratios with 95% confidence intervals (CI) were calculated to assess for differences between groups. We also estimated the cumulative incidence of mortality at six months after TB diagnosis with (corrected mortality estimate) and without (uncorrected mortality estimate) including patients who died prior to TB treatment initiation. We carried out sensitivity analyses of these estimates by considering all patients whose vital status was not ascertained to be either alive or dead. All data analyses were conducted using STATA version 15.1 (Stata Corporation, Texas, USA).

### Ethics statement

This study was approved by the Makerere University School of Medicine Research and Ethics Committee of the College of Health Sciences (Ref: 2016–132) and by the Uganda National Council of Science and Technology.

## Results

From January to June 2018, 510 patients in the ten facilities had a positive Xpert^®^ MTB/RIF test result recorded in the laboratory register. About two thirds of all patients at both primary care facilities (HC IVs) and hospitals were male. A third of all patients diagnosed with TB were HIV co-infected. This proportion was comparable between primary care facilities (HC IV) and hospitals. A hundred (19.6%) patients were not initiated on TB treatment within two weeks of diagnosis (pretreatment LFU). The proportion of patients who experienced pretreatment LFU was higher at hospitals. (Table 1).

**Table 1:** Baseline characteristics of study participants by level of healthcare facility.

Characteristic	Primary-care facility	Hospital
	(HC IV) N = 122	N = 388
**Sex**		
Male	69 (56.6)	243 (62.6)
Female	53 (43.4)	145 (37.4)
**Age**		
15–24	34(27.8)	80 (20.6)
25–34	41(33.6)	123 (31.7)
35–44	22 (18.0)	89 (22.9)
45+	25 (20.5)	96 (24.7)
**HIV status (n = 480)**^β^		
HIV-negative	77 (63.6)	242 (67.4)
HIV-positive	44 (36.4)	117 (32.6)
**Treatment initiation**		
≤2 weeks after diagnosis	106 (86.9)	304(78.4)
>2 weeks after diagnosis (pretreatment LFU)	16(13.1)	84(21.6)
Distance from health facility n = 470)*		
≤5km	42(35.3)	91(25.9)
6-15km	43(36.1)	79(22.5)
16-25km	24(20.2)	63(17.9)
>25km	10(8.4)	118 (33.6)

^**β**^ 30 patients had missing data 1 from primary care facilities (HC IV) and 30 from hospitals

* 40 patients had missing data 3 from primary care facilities (HC IV) and 37 from hospitals

**Fig 1:**
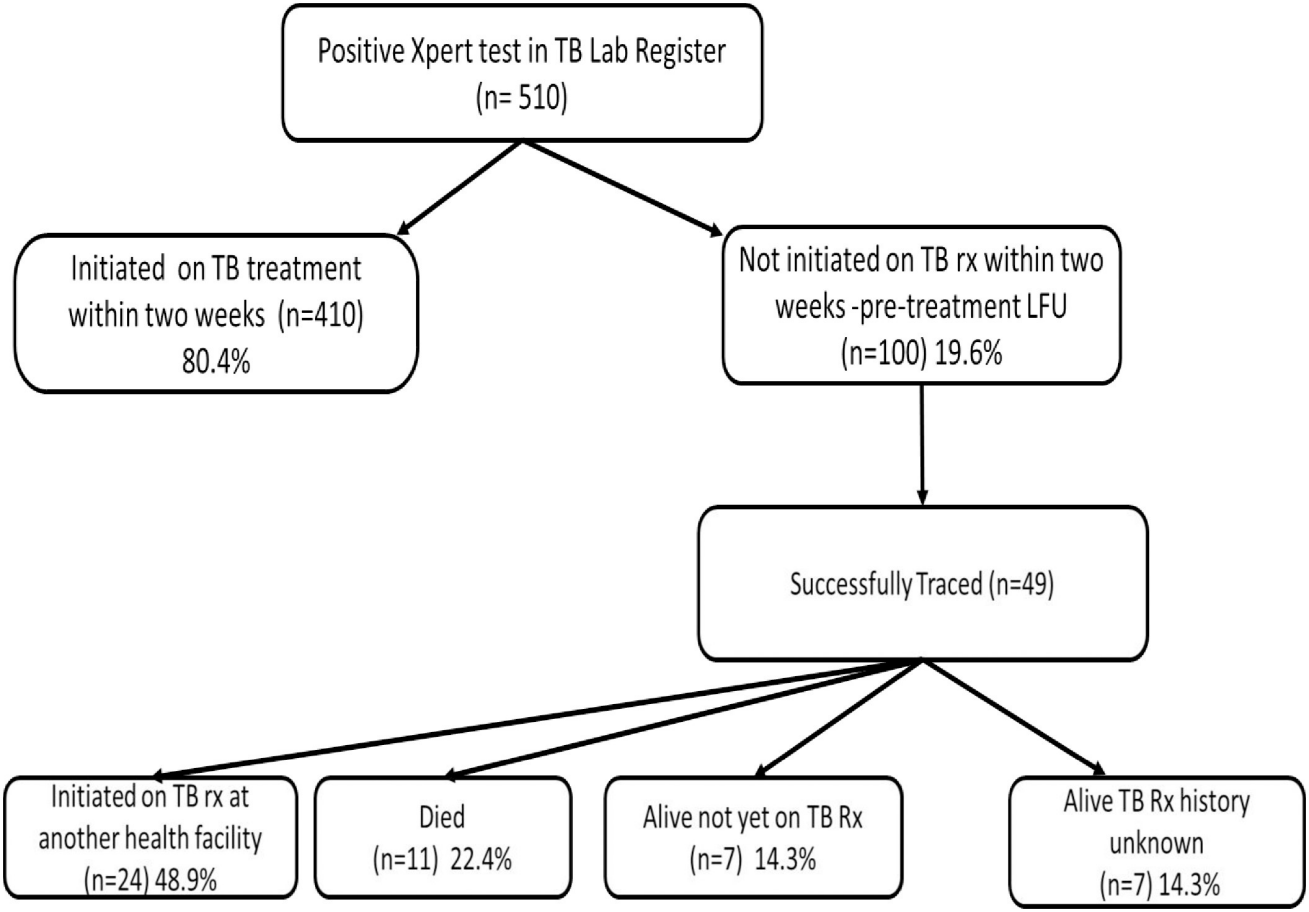
Patient flow chart.

Out of the 100 patients who experienced pretreatment loss to follow-up, we were able to ascertain the vital status of 49 patients: 24 (48.9%) were later initiated on TB treatment in a median time to treatment initiation of 30 (IQR 21–50) days; 11 (22.4%) died prior to treatment initiation in median time of 22 (IQR 11–55) days; 7 (14.3.%) were alive but not on TB treatment, and 7 (14.3%) were confirmed alive by relatives/friends but no treatment information was available (Fig 1).

There were no significant differences in age, sex and HIV status between patients who did and those who did not have their vital status ascertained (Table 2).

**Table 2:** Characetristics of patients who were successfully traced versus those who were not.

Characteristic	Successfully traced	Not Successfully traced	P value (chi2)
	N = 49	N = 51	
**Sex**			
Male	29 (59.8)	28 (54.9)	
Female	20 (40.8)	23 (45.1)	0.67
**Age**			
15–24	12 (24.5)	9 (17.7)	
25–34	14 (28.5)	19(37.3)	0.36
35–44	12 (24.5)	8 (15.7)	0.27
>45	11 (22.5)	15 (29.4)	0.43
**Healthfacility level**			
HC IV	7 (14.3)	9(17.7)	
Hospital	42(85.7)	42(82.4)	0.65
**HIV status (n = 70)**^₽^			
HIV-negative	20 (43.5)	12 (50.0)	
HIV-positive	26 (56.5)	12 (50.0)	0.60
**Distance from healthfacility (n = 77)**^¥^			
≤ 5km	15 (34.1)	16 (17.7)	
6-15km	9 (20.4)	7 (37.3)	0.98
16-25km	8 (18.2)	5(15.7)	0.68
>25km	12 (27.3)	5(29.3)	0.32

^**₽**^ 30 patients had missing data 3 who were successfully traced and 27 who were not successfully traced

^**¥**^ 23 patients had missing data 5 who were successfully traced and 18 who were not successfully traced

Out of the 410 patients who did not experience pretreatment LFU, we were able to establish the vital status of 382; 312 (81.7%) successfully completed treatment, 33 (8.6%) died, 5 (1.3%) failed first line therapy and were started on second line therapy and 32 (8.3%) could not be traced.

### Mortality within six months of diagnosis

Six-month mortality outcomes were for 431 (84.5%) patients, of which 49 did and 382 did not experience pretreatment LFU (Fig 1). There were 11 deaths among the 49 patients who experienced pretreatment LFU and 33 deaths among the 382 patients who did not experience pretreatment LFU. Mortality at six months was higher among patents who experienced pretreatment LFU compared to those who did not (48.1/1000py vs. 22.9/1000py HR 3.18, 95% CI 1.61–6.30).

**Fig 2:**
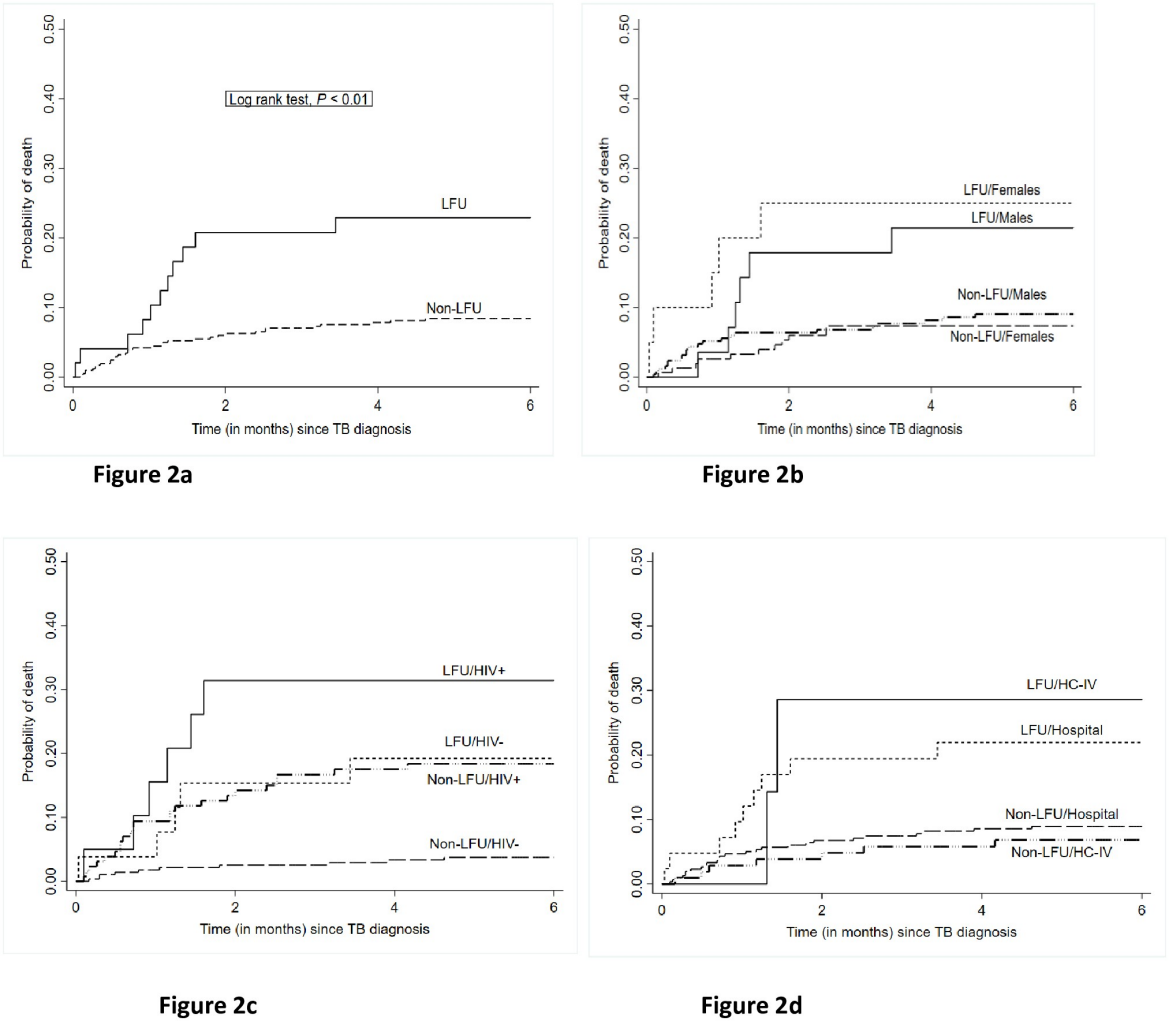
Kaplan Meir curves. Kaplan Meir curves showing cumulative incidence of mortality among patients who did and did not experience pretreatment LFU Fig 2a) overall Fig 2b) stratified by sex Fig 2c) stratified by HIV status and Fig 2d) stratified by health facility level.

In stratified analyses (Table 3), mortality at six months was significantly higher among those who experienced pretreatment LFU in HIV uninfected patients (HR 5.59, 95% CI 1.91–16.34) (Fig 2b); women (HR 4.25, 95% CI 1.48–12.22) (Fig 2c) and patients who received care from hospitals (HR 2.89, 95%CI 1.36–6.18). Mortality at six months was also higher among those who experienced pretreatment LFU for HIV-positive patients and at primary care facilities (HC IVs) but these differences did not reach statistical significance HR. 1.86, 95% CI 0.76–4.58 and HR. 4.52, 95% CI 0.94–21.8 respectively (Fig 2b). Mortality for patients who experienced pretreatment LFU increased with age, particularly among patients 45 years and older where mortality at six months was four times higher (HR 4.28, 95% CI 1.32–13.93) (Table 3).

**Table 3:** Hazard ratios of mortality comparing patients initiated on TB treatment with those who experienced pretreatment loss to follow-up overall and stratified by HIV status.

	LFU (N = 49)		Non LFU (N = 410)		Hazard Ratio (HR) (95% CI)*[LFU/Non LFU]
	No. of deaths	Mortality rates per 1000PY (95% CI)	No. of deaths	Mortality rates per 1000PY (95% CI)	
Overall	11	48.1 (26.6–86.8)	33	22.9 (11.3–22.4)	3.18 (1.61–6.30)
**Stratification**					
**HIV status**					
HIV-positive	6	75.8 (34.0–168.7)	23	38.4 (25.5–57.7)	1.86 (0.76–4.58)
HIV-negative	5	38.0 (15.8–91.2)	10	6.8 (3.7–12.6)	5.59 (1.91–16.34)
**Sex**					
Male	6	43.4 (19.5–96.7)	22	17.4 (11.5–26.4)	2.64 (1.07–6.52)
Female	5	43.4 (19.5–96.7)	11	13.7 (7.6–24.7)	4.25 (1.48–12.22)
**Health facility level**					
HC-IV	2	61.1 (15.3–244.1)	7	12.6 (6.0–26.5)	4.52 (0.94–21.8)
Hospital	9	45.9 (23.9–88.2)	26	17.2 (11.7–25.2)	2.89 (1.36–6.18)
**Age group**					
15–24	1	16.5 (2.33–117.4)	5	10.6 (4.4–25.5)	1.62 (0.19–13.9)
25–34	2	27.6 (6.9–110.2)	9	13.5 (7.0–26.0)	2.64 (0.57–12.22)
35–44	4	79.2 (29.7–211.1)	10	21.9 (11.8–40.7)	3.44 (1.08–10.96)
≥45	4	88.4 (33.2–235.5)	9	18.9 (9.8–36.4)	4.28 (1.32–13.93)

LFU = pre-TB treatment loss to follow-up, Non LFU = Non pre-TB treatment loss to follow-up (also referred to as initiated on treatment).

* Hazard ratios from Cox regression model.

### Correction of mortality estimates

**Fig 3:**
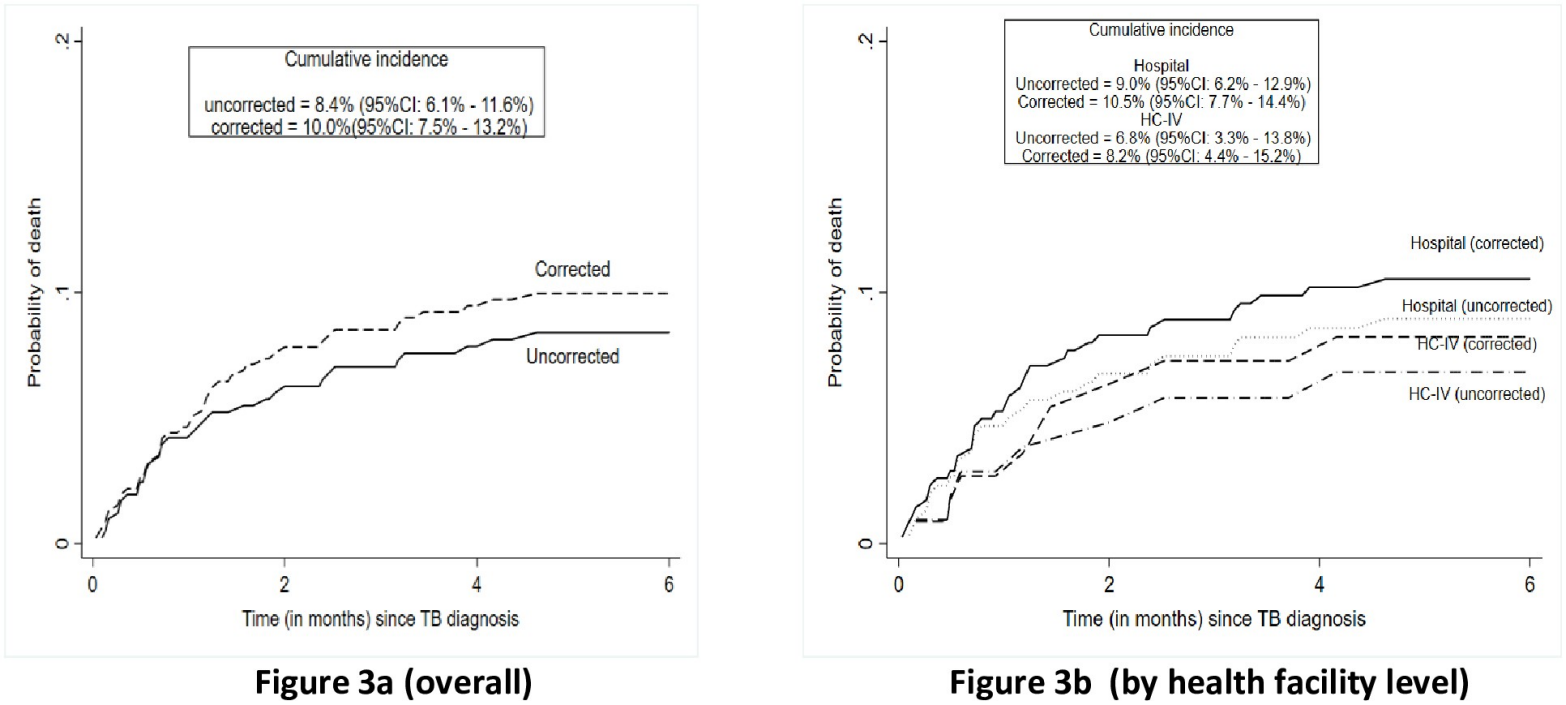
Uncorrected and corrected TB mortality estimate at health facility level 6 months after TB diagnosis. **Uncorrected** indicates cumulative mortality rate among persons started on TB treatment, **corrected** indicates cumulative mortality updated using vital status of patients successfully traced.

After correcting for mortality prior to treatment initation among patients with pretreatment LFU, health facility level mortality estimates increased from 8.4% (95%CI 6.1%-11.6%) to 10.2% (95%CI 7.7%-13.4%) on overall, from 6.8% (95%CI 3.3%-13.8%) to 8.4% (95%CI 4.5%-15.5%) at HC IV level and from 9.0% (95%CI 6.2%-12.9%) to 10.7% (95%CI 7.8%-14.6%) at hospital level (Fig 3). Sensitivity analyses treating all patients whose vital status was not ascertained as alive or dead resulted in updated mortality estimates of 8.6% (95%CI 5.6%-10.4%) and 20.2% (95%CI 16.5%-23.7%), respectively (S2 Fig).

## Discussion

Although a number of studies have described pretreatment LFU in high burden resource limited settings, few studies have traced patients not initiated on TB treatment to determine their outcomes. Our study traced 100 patients who experienced pretreatment LFU to ascertain the effect of pretreatment LFU on TB-associated mortality. We found that the risk of TB associated mortality was almost three times higher among patients who experienced pretreatment LFU compared to those who did not. The risk of death among patients who experienced pretreatment LFU increased if they were female, HIV-negative and belonged to an older age-group.

The majority of deaths (10/11) among patients who experienced pretreatment LFU occurred prior to TB treatment initation and relatively shortly after the diagnosis was made (median 22, IQR 11–55 days). Similar estimates of mortality prior to TB treatment initiation have been reported among patients who experience pretreatment LFU in South Africa (24%) [10] and India (19%;27.6%) [11, 12]. In all studies, these deaths occurred shortly after TB diagnosis implying that patients presented with advanced TB disease and still were not initiated on treatment. In sub-Saharan Africa, a complex interplay between patient and health system factors collude to delay presentation until TB disease is advanced. Patient factors such as lack of knowledge about TB symptoms coupled with stigma associated with TB have been documented to contribute to delayed health seeking. Health system factors particularly the lack of recognition of TB signs and symptoms by healthcare workers results in repeated visits to health facilities before a diagnosis of TB is made and contributes significantly to delayed TB diagnosis [13–15]. Improving outcomes of patients diagnosed with TB should include measures to promote earlier care-seeking through health promotion campaigns. Public health facilities need streamlined procedures to ensure that all patients are screened for TB, those with a positive symptom screen receive a diagnostic test, and if positive, are started on TB treatment.

Among patients whose vital status was ascertained, a higher proportion of HIV-positive patients died prior to treatment initiation (6/20) vs (5/29-for HIV-negative patients). High mortality from HIV-associated TB even in the era of increased ART availability has been documented in other sub-Saharan African settings [16, 17]. In Uganda, a study from the infectious diseases clinic located within Mulago National Referral Hospital found consistently high TB mortality rates (>15%) over seven years of ART scale up [18]. In this study, presentation to care with advanced HIV disease (defined as CD4 cell count <200 cells/ul at enrollment) doubled the risk of mortality from TB. These findings suggest that stronger emphasis should be put on rapid TB treatment initiation for patients co-infected with HIV [19]. In addition to quality improvement initiatives to better track HIV-positive patients along the diagnostic cascade and improve treatment initiation, incorporating point of care tests with shorter turnaround times e.g. lateral flow lipoarabinomannan (LF-LAM) into the diagnostic algorithm for eligible HIV-positive patients may further improve TB treatment initation and reduce mortality [20].

During the six months following TB diagnosis, pretreatment LFU was associated with a three-fold increase in overall TB mortality HR 3.18, (95% CI 1.61–6.30). The increase in mortality was more marked among HIV-negative patients HR 5.59, (95% CI 1.91–16.34), women HR 4.25, (95% CI 1.48–12.22) and patients ≥45years HR 4.28, (95% CI 1.32–13.93). HIV-negative patients when placed on appropriate TB therapy generally have good treatment outcomes with CFRs reported at 3.5% (95% CI 2.0%-4.9%) compared to 18.8% (95% CI 14.8%-22.8%) among HIV-positive patients [21]. Our study highlights the marked negative effect of pretreatment LFU on HIV-negative patients and shows that quality improvement interventions to improve TB care in our setting, which in the recent past have heavily focused on patients co-infected with HIV should be broadened to include HIV-negative patients.

Higher background rates of HIV co-infection among women in our population coupled with financial barriers to accessing healthcare services probably contributed to higher mortality observed among women who experienced pretreatment LFU. In Uganda, HIV prevalence among women is almost twice that among men (7.6% vs 4.7%) [22] and in our cohort 40.1% (73/182) of women were co-infected with HIV compared to 29.5% (88/299) of men pvalue 0.02. In addition, the recently concluded Uganda TB patients’ costs survey found that women are more likely to incur catastrophic financial costs (defined as ≥20% of annual household income) while seeking care for TB [23] which are increased by the need to make repeated visits to health facilities before a TB diagnosis is made and/or treatment is initiated. Ensuring that patients are screened for TB and started on TB treatment as soon as they present to the healthcare system (same-day diagnosis) may reduce pretreatment LFU and in turn improve outcomes among women diagnosed with TB. Increased mortality among older patients with TB has been reported in other studies [24, 25] and may be caused by late diagnosis due to atypical presentations and presence of other co-morbidities.

Correction of health facility level mortality estimates to include deaths prior to treatment initiation resulted in an overall increase in cumulative TB mortality from 8.4% (95%CI 6.1%-11.6%) to 10.2% (95%CI 7.7%-13.4%). These results still show that the use of uncorrected estimates in routine program reporting may lead to an underestimation of TB mortality.

## Limitations

This was a retrospective study that used program data. Challenges with missing data and variable data quality affected our ability to follow up all patients. Importantly, vital status could not be ascertained for 51% of patients who experienced pretreatment LFU. Many patients could not be traced because of inaccurate address information and/or unreachable phone contacts. Although there were no significant differences in age, sex, and HIV status between patients who did and did not have vital status ascertained, it is likely that mortality was even higher among those who could not be traced [8, 12]. Nonetheless, our sensitivity analysis reports the full range of possible mortality estimates.

## Conclusion

Patients with confirmed TB who experience pretreatment LFU have high mortality within the first six months. Efforts should be made to prioritise linkage to treatment for this group of patients. Efforts should also be made to incorporate deaths prior to treatment initation when reporting health facility estimates of TB mortality in order to more accurately reflect the health impact of TB.

## Supporting information

S1 File (CSV)

S1 Fig Protocol for patient tracing. (DOCX)

S2 Fig Sensitivity analyses for TB mortality estimates after six months. Cumulative incidence: A) Uncorrected = 8.4%, C) Updated analysis = 10.2%, D) Unsuccessfully traced treated as alive = 9.0%, **E**) Unsuccessfully traced treated as dead = 20.
(DOCX)

## References

[1] World Health Organization. WHO End TB Strategy [Internet]. WHO. 2015 [cited 2019 Jan 21]. p. 16. https://www.who.int/tb/post2015_strategy/en/

[2] World Health Organization. Global Tuberculosis Report 2019 [Internet]. 2019. 278 p. https://www.who.int/tb/publications/global_report/en/

[3] Dodd PJ. Gtbr2018_Online_Technical_Appendix_Global_Disease_Burden_Estimation. 2018;(September).

[4] Korenromp EL, Bierrenbach AL, Williams BG, Dye C. The measurement and estimation of tuberculosis mortality. [State art Ser Tuberc Ed by ID Rusen Number 5 Ser Int J Tuberc Lung Dis 2009. 2009;3 1;13(3 19275787

[5] MacPherson P, Houben RMGJ, Glynn JR, Corbett EL, Kranzer K. Pérdida de seguimiento antes del tratamiento de pacientes con tuberculosis en países de ingresos medios y bajos y en países con carga alta: Una revisión sistemática y metanálisis. Bull World Health Organ. 2014;92(2):126–38. 24623906 10.2471/BLT.13.124800 PMC3949536

[6] Davis JL, Katamba A, Vasquez J, Crawford E, Sserwanga A, Kakeeto S, et al Evaluating tuberculosis case detection via real-time monitoring of tuberculosis diagnostic services. Am J Respir Crit Care Med. 2011;184(3):362–7. 10.1164/rccm.201012-1984OC 21471088 PMC3175538

[7] Manabe YC, Zawedde-Muyanja S, Burnett SM, Mugabe F, Naikoba S, Coutinho A, et al Rapid improvement in passive tuberculosis case detection and tuberculosis treatment outcomes after implementation of a bundled laboratory diagnostic and on-site training intervention targeting mid-level providers. Open Forum Infect Dis. 2015;2(1). 10.1093/ofid/ofv030 26034778 PMC4438908

[8] Squire SB, Belaye AK, Kashoti A, Salaniponi FML, Mundy CJF, Theobald S, et al “Lost” smear-positive pulmonary tuberculosis cases: Where are they and why did we lose them? Int J Tuberc Lung Dis. 2005;9(1):25–31. 15675546

[9] Mugauri H, Shewade HD, Dlodlo RA, Hove S, Sibanda E. Bacteriologically confirmed pulmonary tuberculosis patients: Loss to follow-up, death and delay before treatment initiation in Bulawayo, Zimbabwe from 2012–2016. Int J Infect Dis [Internet]. 2018;76:6–13. Available from: 10.1016/j.ijid.2018.07.012 30030177

[10] Botha, Den Boon S, Verver S, Dunbar R, Lawrence KA, Bosman M, et al Initial default from tuberculosis treatment: How often does it happen and what are the reasons? Int J Tuberc Lung Dis. 2008;12(7):820–3. 18544210

[11] Communication S. Initial default among cases diagnosed in the community survey and at the health facilities. Indian J Tuberc. 2005;52:153–6.

[12] Thomas BE, Subbaraman R, Sellappan S, Suresh C, Lavanya J, Lincy S, et al Pretreatment loss to follow-up of tuberculosis patients in Chennai, India: A cohort study with implications for health systems strengthening. BMC Infect Dis. 2018;18(1). 10.1186/s12879-018-3039-3 29587651 PMC5872574

[13] Shete PB, Haguma P, Miller CR, Ochom E, Ayakaka I, Davis JL, et al Pathways and costs of care for patients with tuberculosis symptoms in rural Uganda. Int J Tuberc Lung Dis. 2015;19(8):912–7. 10.5588/ijtld.14.0166 26162356 PMC6602531

[14] Uganda Ministry of Health. The Republic of Uganda The Uganda National Tuberculosis Prevalence Survey, 2014–2015 Survey Report [Internet]. 2015. http://health.go.ug/content/uganda-national-tuberculosis-prevalence-survey-2014-2015-survey-report),

[15] Zawedde-Muyanja S, Manabe YC, Sewankambo NK, Nakiyingi L, Nakanjako D. Xpert^®^ MTB/RIF associated with improved treatment initiation among patients with smear-negative tuberculosis. Int J Tuberc Lung Dis. 2018;22(12):1475–80. 10.5588/ijtld.17.0460 30606320 PMC6306042

[16] Le NAT, Bui TQ, Nguyen BH, Nguyen CK, Bui HTT, Nguyen N V. The Situation of Mortality and Burden of Tuberculosis at Some Hospitals in Vietnam in 2015. In: C54 TUBERCULOSIS EPIDEMIOLOGY. American Thoracic Society; 2019. p. A5162–A5162.

[17] Ngari M, Abdullahi O, Sanga D, Katana G, Willetts A. PO 8417 Rising trends in TB mortality amid decline in cases notified in a rural county in kenya: cohort study. Br Med J [Internet]. 2019;4(3). Available from: 10.1136/bmjgh-2019-EDC.89

[18] Musaazi J, Sekaggya-Wiltshire C, Kiragga AN, Kalule I, Reynolds SJ, Manabe YC, et al Sustained positive impact on tuberculosis treatment outcomes of TB-HIV integrated care in Uganda. Int J Tuberc Lung Dis. 2019;23(4):514–21. 10.5588/ijtld.18.0306 31064632

[19] Tenforde MW, Walker AS, Gibb DM, Manabe YC. Rapid antiretroviral therapy initiation in low- and middle-income countries: A resource-based approach. PLoS Med. 2019;16(1):1–10. 10.1371/journal.pmed.1002723 30645592 PMC6333330

[20] Storla DG, Yimer S, Bjune GA. A systematic review of delay in the diagnosis and treatment of tuberculosis. BMC Public Health. 2008;8:15 10.1186/1471-2458-8-15 18194573 PMC2265684

[21] Straetemans M, Glaziou P, Bierrenbach AL, Sismanidis C, van der Werf MJ. Assessing tuberculosis case fatality ratio: A meta-analysis. Vol. 6, PLoS ONE. 2011 10.1371/journal.pone.0020755 21738585 PMC3124477

[22] Uganda Ministry of Health. Uganda Population-Based HIV Impact Assessment. 2017;(August 2017):62–5.

[23] Uganda Ministry of Health. Direct and Indirect costs due to Tuberculosis and proportion of Tuberculosis-affected households experiencing catastrophic costs due to TB in Uganda [Internet]. 2019. health.go.ug/sites/default/files/Tuberculosis patients cost survey Report_2019.pdf

[24] Loveday M, Mzobe YN, Pillay Y, Barron P. Figures of the dead: A decade of tuberculosis mortality registrations in South Africa. S Afr Med J. 2019;109(10):728–32. 10.7196/SAMJ.2019.v109i10.14073 31635566

[25] Negin J, Abimbola S, Marais BJ. Tuberculosis among older adults—time to take notice. Int J Infect Dis. 2015;32:135–7. 10.1016/j.ijid.2014.11.018 25809769

