# Supplementary figures and images for: Estimating the effect of pretreatment loss to follow up on TB associated mortality at public health facilities in Uganda
Source: PLoS One. 2020 Nov 18;15(11):e0241611. doi: 10.1371/journal.pone.0241611 (PMC7673517; doi:10.1371/journal.pone.0241611)

**S1 Fig: Protocol for patient tracing**

**
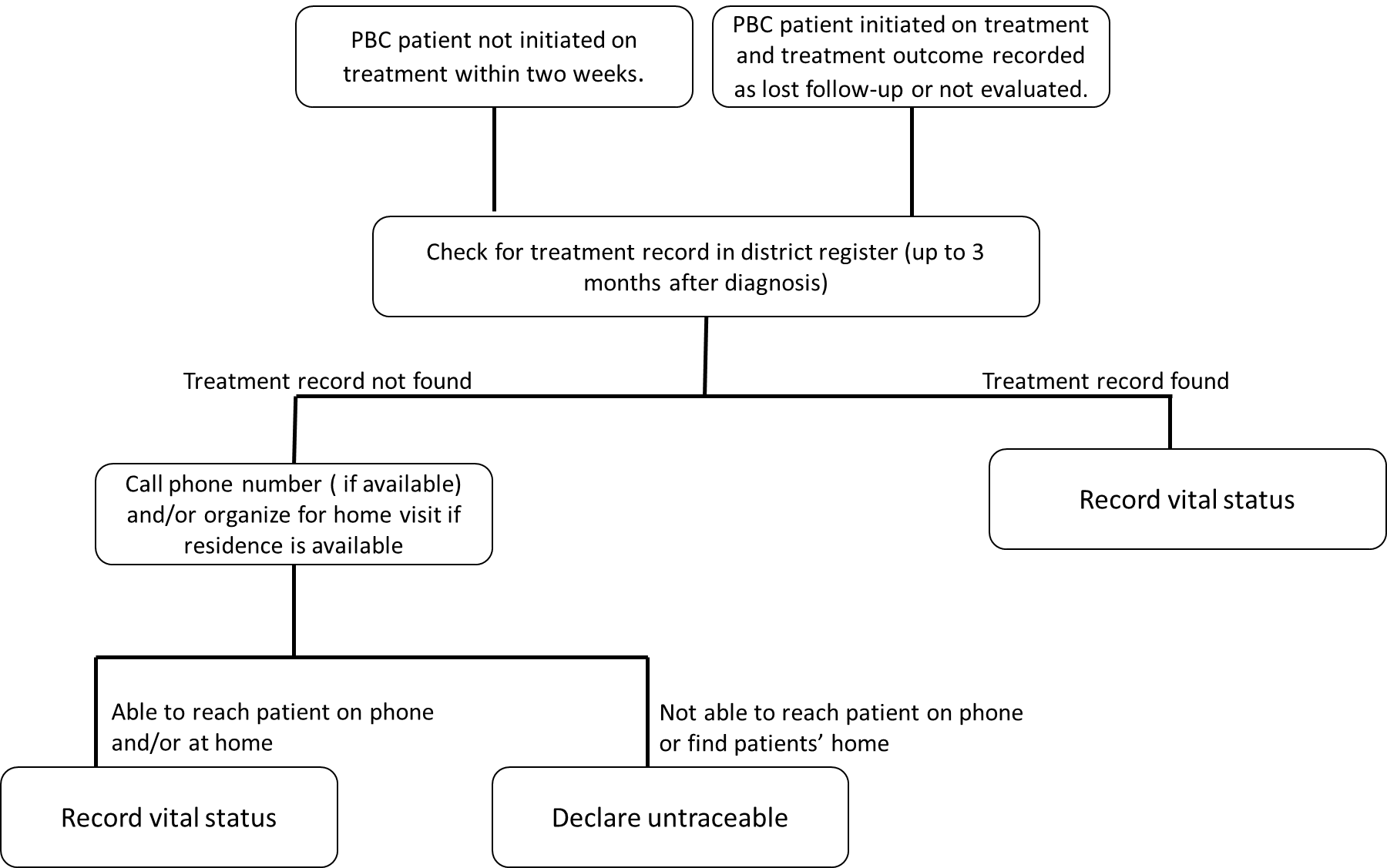
**

Supplement: S1 Fig — (DOCX) [file pone.0241611.s002.docx]
