# Supplementary material for: Estimating the effect of pretreatment loss to follow up on TB associated mortality at public health facilities in Uganda
Source: PLoS One. 2020 Nov 18;15(11):e0241611. doi: 10.1371/journal.pone.0241611 (PMC7673517; doi:10.1371/journal.pone.0241611)
Supplement: S2 Fig — Cumulative incidence: A) Uncorrected = 8.4%, C) Updated analysis = 10.2%, D) Unsuccessfully traced treated as alive = 9.0%, E) Unsuccessfully traced treated as dead = 20. (DOCX) [file pone.0241611.s003.docx]

**S2 Fig: Sensitivity Analyses for TB mortality estimates over 6 months**

**
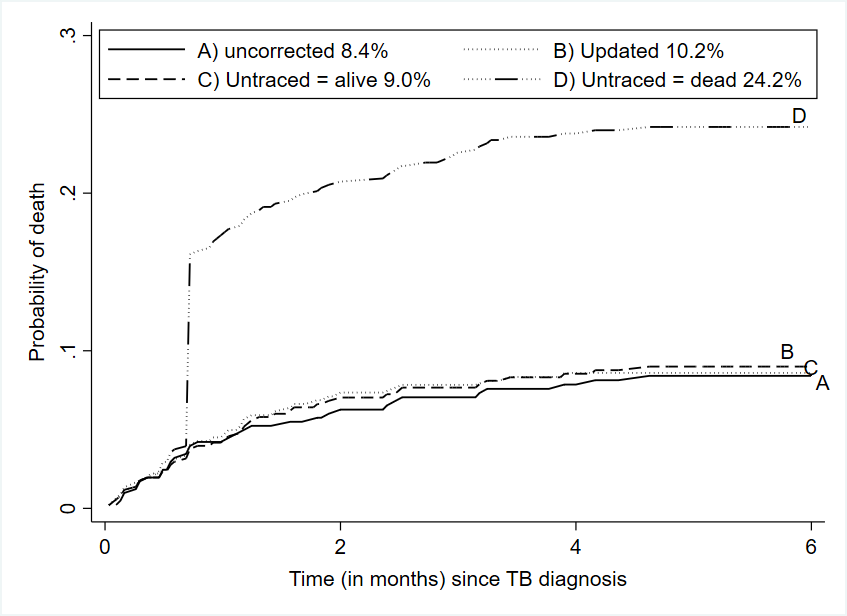
**

***Cumulative incidence proportion****:* ***A****) Uncorrected = 8.4%,* ***C****) Updated analysis = 10.2%,* ***D****) Unsuccessfully traced treated as alive = 9.0%,* ***E****) Unsuccessfully traced treated as dead = 24.2%*
